# Small Natural Cyclic Peptides from DBAASP Database

**DOI:** 10.3390/ph17070845

**Published:** 2024-06-27

**Authors:** Evgenia Alimbarashvili, Natia Samsonidze, Maia Grigolava, Malak Pirtskhalava

**Affiliations:** Ivane Beritashvili Center of Experimental Biomedicine, Tbilisi 0160, Georgia; samsonidze.natia97@gmail.com (N.S.); maia.grigolava@science.org.ge (M.G.)

**Keywords:** antimicrobial peptides, AMPs, cyclic peptides, macrocyclization

## Abstract

Antimicrobial peptides (AMPs) are promising tools for combating microbial resistance. However, their therapeutic potential is hindered by two intrinsic drawbacks—low target affinity and poor in vivo stability. Macrocyclization, a process that improves the pharmacological properties and bioactivity of peptides, can address these limitations. As a result, macrocyclic peptides represent attractive drug candidates. Moreover, many drugs are macrocycles that originated from natural product scaffolds, suggesting that nature offers solutions to the challenges faced by AMPs. In this review, we explore natural cyclic peptides from the DBAASP database. DBAASP is a comprehensive repository of data on antimicrobial/cytotoxic activities and structures of peptides. We analyze the data on small (≤25 AA) ribosomal and non-ribosomal cyclic peptides from DBAASP according to their amino acid composition, bonds used for cyclization, targets they act on, and mechanisms of action. This analysis will enhance our understanding of the small cyclic peptides that nature has provided to defend living organisms.

## 1. Introduction

Numerous endeavors are underway to explore natural peptides as prototypes for novel drugs. For instance, antimicrobial peptides (AMPs) are considered hopeful tools to solve the problem of resistance [1,2]. Along with antimicrobial properties, AMPs possess anticancer activities [3], cell-penetrating abilities [4], immune-system-modulating capabilities [5], and other biologically significant properties [6], majorly attributed to their common feature of membrane targeting. However, in some cases, their activities rely on interactions with membrane or cytoplasmic proteins. Peptide-based drugs predominantly work by inhibiting interactions between large surfaces, such as protein–protein, protein–nucleic acids, protein–lipid, and lipid–lipid interactions. Additionally, there are small-molecular-weight peptide-based drugs that inhibit interactions with specific enzymes or receptors. Despite their potential, the current design process for novel peptide-based drugs remains challenging. The main hurdle lies in designing druggable peptides [7].

Drugs need to be formulated to resist rapid chemical destruction in living organisms and to be easily absorbed and distributed. For traditional small-molecular-weight drugs, druggability is determined by the Rule of Five (Ro5) [8], which defines limits for molecular size (MW < 500 Da) and polarity. These limitations in drug discovery have constrained the range of targets, mostly limited to the active sites of enzymes and receptors, that can be effectively inhibited by small molecules [9]. Indeed, there are small-molecular-weight ultra-short (<6 AA) peptide-based drugs, such as β-lactam antibiotics, that inhibit interactions with specific enzymes or receptors. The small size of traditional drugs makes it challenging for them to effectively inhibit large surface interactions. Consequently, there is a broad category of target sites presented by large surfaces and referred to as ‘undruggable’ binding sites [10,11,12].

The unique physicochemical properties of short (6–25 AA) peptide-based drugs, including their larger size and more flexible backbone, enable them to act as potent inhibitors of ‘undruggable’ binding sites. However, it is worth noting that peptides may face two inherent limitations: membrane impermeability and poor in vivo stability. To address these issues, macrocyclization can improve the pharmacological properties and bioactivity of peptides. Notably, half of the beyond-Rule-of-Five (bRo5) drugs that target flat or groove-like interfaces are macrocycles [11,12], many of which originate from natural product scaffolds and present as peptide-based [13]. Consequently, macrocyclic peptides emerge as promising candidates for drug development, deserving significant attention.

Macrocyclic peptides constitute a highly diverse family of peptides found in various organisms, including bacteria, fungi, and protists. Recent studies on natural and synthetic model systems have demonstrated the increasing utilization of cyclic peptide natural products in designing bRo5 molecules, which exhibit exceptional cell permeability and oral absorption [14]. Macrocyclization is employed to enhance metabolic stability. To achieve cell permeability, macrocycles must possess a ‘chameleonic’ ability, allowing them to change their conformation to expose polar groups in an aqueous solution while concealing them during traversal of lipid membranes [15].

Small macrocyclic peptides (ultra-short and short) represent more valuable drug candidates. The Database of Antimicrobial Activity and Structure of Peptides (DBAASP) is a comprehensive repository for small cyclic peptides [16], freely accessible at www.dbaasp.org (accessed on 4 June 2024). Currently, the database contains information on 597 non-ribosomal and 1413 ribosomal cyclic peptides (data were obtained on 13 May 2024. The length of non-ribosomal cyclic peptides does not exceed 25 amino acids (AA), making them small peptides. Additionally, the number of ribosomal cyclic peptides of a length less than 26 AA equals 484. In this review, we will explore small cyclic peptides of DBAASP that have been offered by nature to defend living organisms. The volume of DBAASP allows us to uncover common peculiarities of the cyclic structures formed in natural peptides.

## 2. Definition of Intrachain Bonds and Rings of Natural Cyclic Peptides

Various kinds of intrachain bonds, such as amide (AMD), ester (EST), thioether (TIE), amine (AMN), etc., are used to cyclize natural peptides (see Table 1). As a result, many kinds of rings are created.

In general, a certain kind of ring, for example, lactam (LAC), can be formed by different types of bonds and three kinds of chain linking: sidechain-to-sidechain (SSB), sidechain-to-mainchain (SMB), and mainchain-to-mainchain (MMB). Consequently, in this work, each ring formed by a specific intrachain bond has been characterized by the following three parameters: the type of bond (AMD, for example), the chains participating in the bonding (SMB, for example), and the type of ring formed (LAC, for example). In some cases, special names are employed for widespread types of rings to emphasize the differences between rings formed by similar bonds. For instance, a peptide is a polymer of amino acids with a mainchain composed of a string of amide bonds. Consequently, the majority of rings formed by intrachain bonds constitute macrolactams. Amide bonds formed between the N-terminal amine and C-terminal carboxyl groups create a macrolactam ring and can be defined by the following three parameters (AMD, MMB, and LAC). An analogous macrolactam ring formed by bonding between sidechain amine (for example, of Lys) and C-terminal carboxyl is presented in the three-parameter definition as (AMD, SMB, and LAC). To emphasize that the first macrolactam corresponds to the case of head-to-tail linking, the ring has been renamed as NCB. Therefore, a ring of head-to-tail cyclized peptides in the three-parameter definition is presented as (AMD, MMB, and NCB) instead of the above-mentioned (AMD, MMB, and LAC). Another example involves lactams formed in cyclic dipeptides, where the ring name diketopiperazine (DKP) is used instead of lactam.

Thus, rings closed by different kinds of bonds between pairs of residues spaced by one or more amino acids can be defined as lactams (macrolactams and macrocyclic amides). In this work, lactams have been classified based on bonds that close them (Table 1). Above, examples of rings closed by amide bonds, such as NCB and DKP, have been presented. As an additional example, the rings closed by ester bonds between carboxyl groups of sidechains (for example, of Asp or Glu) and C-termini of peptides can be considered. This type of ring can be simultaneously classified as both a lactam and a lactone (cyclic ester). However, for more specificity, it has been defined as LCN (lactone). All bonds used for ring closing in small natural peptides, along with the names of corresponding rings as used in DBAASP [16] and in this work, are presented in Table 1.

## 3. Small Cyclic Peptides of DBAASP

DBAASP is a manually curated database that offers detailed information on the physicochemical and structural features, as well as antimicrobial and cytotoxic activities, of peptides assessed in vitro. The current version of the database contains data on more than 22,000 peptides, the antimicrobial/cytotoxic potency of which has been assessed in vitro [16]. DBAASP provides information on peptides belonging to both Ro5 and bRo5 groups. In terms of size, peptides can be classified into two types: those with a length of ≤5 AA, referred to as ultra-short peptides (USPs), and those with a length of 6–25 AA, referred to as short peptides (SPs).

### 3.1. Ultra-Short Cyclic Peptides

The number of natural USPs (peptides with a length of ≤5 AA) in DBAASP is 275, comprising 7 ribosomal and 268 non-ribosomal peptides. Among these, there are 232 ultra-short cyclic peptides (USCPs), including 230 non-ribosomal and 2 ribosomal peptides. The numbers of USCPs of different lengths and intrachain bonds are presented in Table 2.

**Amide bond.** According to Table 2, rings of non-ribosomal USCPs are mainly (159 peptide) closed by amide bonds. Among these, 147 are closed by mainchain-to-mainchain amide bonds, including 65 NCB and 82 DKP. In 12 peptides containing an LAC ring, the rings are closed by amide bonds between the sidechain of ornithine or Trp and C-terminal carboxyl group.

**Amine and disulfide bonds.** A lactam ring can also be formed by amine bonds between the amine group of mainchain and the β-carbon of sidechain of Cys, as observed in the case of peptides containing a β-lactam ring (see Table 2).

**Figure 1:**
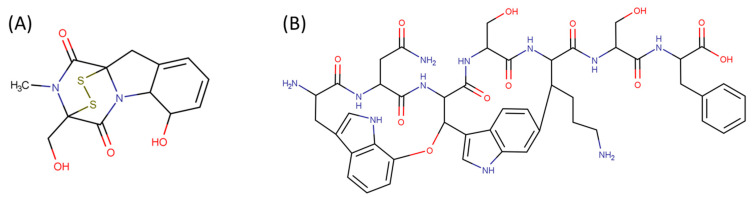
Chemical structures of Gliotoxin (**A**) and Darobactin A (**B**).

Amine bonds also participate in the formation of five-membered saturated pyrrolidine ring in the exotic peptide, Gliotoxin (DBAASPN_21167). Gliotoxin is a unique non-ribosomal dipeptide in DBAASP, containing three different rings, including a disulfide bond (see Figure 1). In this peptide, the disulfide bond links two α-carbon atoms (creating DSL ring) and so it differs from other disulfide-bonded peptides in DBAASP, which are created through disulfide bonding between Cys residues (CST ring). It is worth noting that Gliotoxin is also cyclized by a head-to-tail amide bond and, therefore, constituting a diketopiperazine ring.

CST rings, created through disulfide bonding between Cys residues, appear only in three non-ribosomal peptides: PM181110 (DBAASPN_21712), Malformin E (DBAASPN_21150), and Malformin A1 (DBAASPN_21151).

**Ester bond.** In 65 USCP, comprising only non-ribosomal peptides, LCN rings are closed by ester bonds (EST) through the binding between the sidechain of Thr or N-terminal hydroxycarboxylic acids (such as 5-hydroxy-2,4-dimethyl-3-oxooctanoic acid, 2-hydroxy-3-methylbutyric acid, 3-hydroxy-2-methyloctynoic acid, 3-hydroxy-2,4-dimethyldecanoic acid, 3-hydroxy-2,4,6-trimethyldecanoic acid, 2-hydroxy-4-methylpentanoic acid, 2,6,10,14-methyl-3-hydroxy-elcosadienoic acid, etc.) with the carboxyl group of the C-terminal amino acid.

**Thioether (Sulfide) bond.** Thioether bonds (TIE) between the sidechains of neighboring Cys and Val residues close five- or six-membered rings of varying saturation. Saturated five-membered rings are named thiazolinidine (THZD) and occur in β-lactam antibiotics of the penicillin or methicillin family (DBAASPN_20466, DBAASPN_20468, and DBAASPN_20907). In Cephalosporin C (DBAASPN_20467), the cyclized structure is presented by an unsaturated six-membered thiazine (TZ) ring.

Thus, non-ribosomal USCPs are primarily cyclized by amide and ester bonds, while other types (DSB, TIE, and AMN) are used rarely. Among USCPs, only two are ribosomal and both use AMD bonds for cyclization (refer to Table 2).

**Figure 2:**
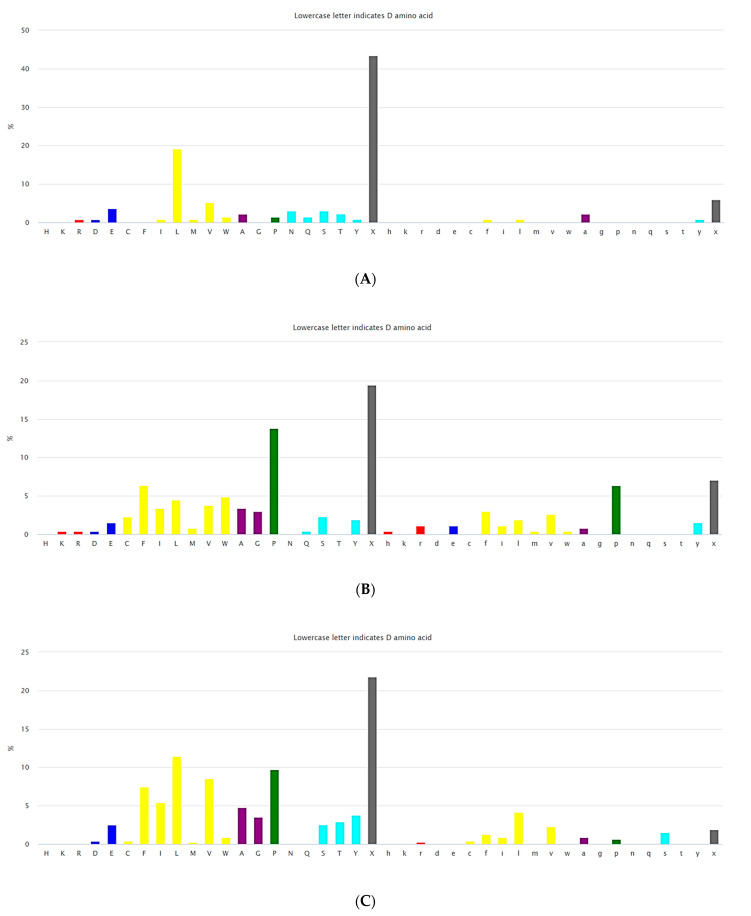
(**A**) The composition of amino acids for non-ribosomal linear peptides of length 2–5. (https://dbaasp.org/statistics?page=compositional-statistics, accessed on 13 May 2024). (**B**) The composition of amino acids for non-ribosomal cyclic peptides of length 2–3. (https://dbaasp.org/statistics?page=compositional-statistics, accessed on 13 May 2024). (**C**) The composition of amino acids for non-ribosomal cyclic peptides of length 4–5. On the *x*-axis, residues are represented in one letter code. Colors: red—positively charged; blue—negatively charged; yellow—hydrophobic; purple—small; green—special; cyan—polar uncharged residues; grey—unusual. (https://dbaasp.org/statistics?page=compositional-statistics, accessed on 13 May 2024).

Natural USCPs rarely use disulfide bonds (DSB) for cyclization. Cys is a scarce amino acid in non-ribosomal linear USPs, as depicted in Figure 2A. Non-ribosomal peptides are rich in unusual amino acids (Figure 2). In contrast to ultra-short linear peptides, USCPs are rich in Trp, Phe, and Pro (Figure 2B,C). Pro is especially abundant in the cyclic dipeptides and tripeptides. This fact can be explained by the necessity of Pro to cyclize ultra-short peptides, as it has the propensity to promote sharp turns in the mainchain of the polypeptide. For example, it has been shown that cyclic tripeptides contain at least one cis peptide bond, while tetrapeptides are the smallest cyclic peptides that can adopt an all-trans conformation, although they generally adopt alternating cis/trans conformation [17]. Consequently, with the lengthening of peptide chains, the sharp need for Pro in cyclization diminishes.

**Figure 3:**
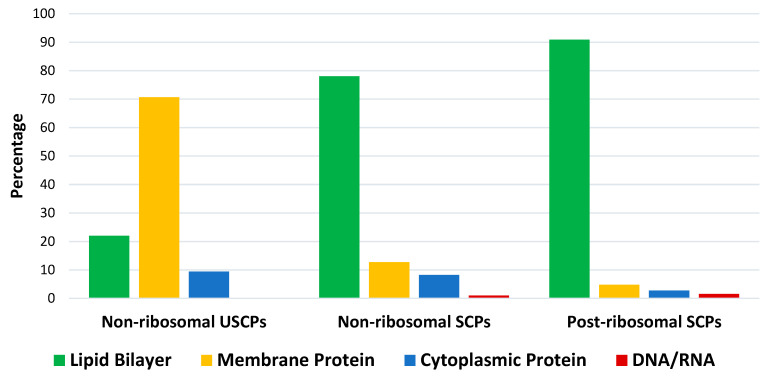
The distribution of peptides acting on specific cellular components (target objects): lipid bilayer, membrane protein, cytoplasmic protein, and DNA/RNA for non-ribosomal USCPs and non- and post-ribosomal SCPs from DBAASP. USCPs—ultra-short cyclic peptides (1–5 AA), SCPs—short cyclic peptides (6–25 AA); analysis performed on 13 May 2024.

The results of the analysis indicate that natural USCPs are mainly synthesized non-ribosomally, predominantly by fungi (131 peptides) and bacteria (102 peptides); only seven peptides originate from archaea, one from Animalia, and two from plantae. They act on a variety of cells, organisms, or viral particles (cancer cells, fungi, Gram-positive and Gram-negative bacteria, viruses, and parasites). Notably, their target objects majorly are proteins (membrane or cytoplasmic, see Figure 3). This observation is explainable. USCPs have the ability to satisfy the Rule of Five and so have druggable targets, enzymes, or receptors. For example, beta-lactam antibiotics targeted the enzyme responsible for cell wall biosynthesis. They have a common core skeleton composed of an enclosed dipeptide formed by the condensation of l-cysteine and d-valine [18]. While, for the head-to-tail cyclized dipeptides, the core skeleton of which consist of 2,5-diketopiperazine ring, it is suggested that they inhibit regulators of bacterial cell population density (‘quorum sensing’) [19,20]. For some members of cyclic dipeptides (for example, cyclic (l-Pro—l-Leu; l-Ser—l-Trp; l-Tyr—l-Pro)), quorum sensing activities have been studied [19]. For other 2,5-diketopiperazines, constituted by hydroxyprolines, an ability to stimulate cytokines or chemokines in macrophages have been supposed [5]. We have to note that, as a rule, USCPs are not membrane-active peptides (see Figure 3).

### 3.2. Short Cyclic Peptides

In contrast to USCPs, a membrane (lipid bilayer) is a main target for short cyclic peptides (SCPs) (see Figure 3). Therefore, they are mainly membrane-active peptides (MAP). It is worth noting that, in addition to the membrane, there are other ‘undruggable’ binding sites targeted by SCPs (Figure 3). The MWs of short peptides (SPs) exceed 600 Da and they are categorized as beyond-Rule-of-Five compounds. They can interact with ‘undruggable’ binding sites and would be considered as a basis for the development of beyond-Rule-of-Five (bRo5) drugs [11]. SPs can be considered druggable if they are capable of changing the surface accessibility of polar groups during the transition from water to the membrane [15]. An additional requirement is resistance to chemical destruction, which can be achieved through cyclization. Consequently, it is interesting to explore the types of natural SCPs that occur.

DBAASP stores data on 1565 ribosomal and 464 non-ribosomal SPs, which are peptides with length in the interval of 6–25 AA. Among these, 482 ribosomal and 367 non-ribosomal peptides are cyclized by intrachain covalent bonds.

#### 3.2.1. Peptides Could Interact with Several ‘Undruggable’ Binding Sites

‘Undruggable’ binding sites can include surfaces of the proteins, DNA, RNA, lipids, etc. Often, undruggable binding sites can consist of surfaces of two or more molecules. For example, in the case of membrane proteins, the binding site can constitute surfaces of the protein and lipids simultaneously. Darobactin (DBAASPR_17389) exclusively targets the bacterial insertase BamA6—the central unit of the BAM [21], while macrocyclic beta-hairpin-shaped peptide JB-95 interacts with both outer membrane and BamA [22].

In general, if a molecule is a membrane protein, instances where there is a distinct binding site on the protein surface, fully separated from the lipid environment, are rare. Moreover, if the targets of a peptide are inside of the cell (such as cytoplasmic proteins, DNA, RNA, ribosome, mitochondrial membrane, etc.), it has to translocate through the lipid bilayer in an energetically dependent or independent manner and so has to interact with the cytoplasmic membrane. Consequently, it is clear why the majority of AMPs are considered membrane-active peptides (MAP).

#### 3.2.2. Non-Ribosomal Short Cyclic Peptides

Various kinds of intrachain bonds, such as amide, ester, thioether, amine, etc., are used to cyclize non-ribosomal SCPs (see Table 3). As a result, many kinds of rings are created. The number and size of rings in peptides also vary. The majority of non-ribosomal cyclic peptides consist of non-proteinogenic amino acids and have modifications on termini. Only 24 non-ribosomal SCPs of DBAASP consist exclusively of proteinogenic amino acids and do not have modification on their C- or N-termini.

As mentioned above, DBAASP stores data on 465 non-ribosomal SPs, including 368 cyclic peptides.

##### Intrachain Bonds and Created Rings in Non-Ribosomal Peptides

**Amide bonds.** Among non-ribosomal SCPs, more than half (204 peptides) have been cyclized by amide bonds (Table 3).

Rings created by amide bonds can be divided into two groups. The number of peptides with rings created by linking terminal amines of the mainchain to the terminal carboxyl of the mainchain, that is NCB, equals 138. In another peptide group (66 peptides), rings are closed by amide bonds between sidechain amine groups and C-terminal carboxyl groups of the mainchain, forming LAC rings (Table 3). For instance, amide bonds between the amine group of the sidechains of 2,4-diaminobutyrate (DAB), Lys, or 2,3-diaminopropionic acid (DAP) or N-terminal 3-aminotetradecanoic acid and mainchain carboxyl groups are cyclized into LAC rings.

**Ester bond.** Apart from amide bonds, other sidechain-to-mainchain bonds are also used to cyclize non-ribosomal SCPs. For example, ester bonds (ESTs) between the sidechains of Ser, Homo Ser, dimethyl-serine, Trh, Allo-Thr, N-methyl-Trh, Tyr, N-terminal hydroxy-fatty acids, and C-terminal carboxyl groups form rings in 142 SCPs defined as lactone (LCN).

**Thioether (sulfide), amine, and carbon bonds.** Non-ribosomal SCPs closing their rings by amine, thioether, and carbon bonds are also presented in the DBAASP, however, in limited numbers. Their scarcity can be explained by many various factors, including a lack of sufficient data in DBAASP at the current moment. Therefore, it would be difficult to make some generalizations about their exoticism. We will only have to describe them.

Amino acid Cys can participate in the creation of intrachain thioether (sulfide) bonds, resulting in the formation of various kinds of rings. Thioether bonds between the sidechain of Cys with the mainchain carbonyl group of neighboring amino acids form an unsaturated thiazoline (THZN) ring in the SCPs of the Bacitracin family (DBAASPN_17106–DBAASPN_17109). Meanwhile, in the SCPs of Pagoamide (DBAASPN_18305), Haligramide (DBAASPN_21016, DBAASPN_21017), and Bisebromoamide (DBAASPN_18509) families, bonding between similar groups of atoms cyclize chain into more aromatic (unsaturated) thiazole (THZ) rings. The same kinds of bonds in Lugdunin (DBAASPN_13304) create a saturated thiazolidine ring.

LAC ring can also be formed through amine bonds between the sidechain carbons of 2,3-diaminoacrylic acids and mainchain amine groups, as observed in peptides of the Callyaerin (DBAASPN_9851-9856) family. Amine bonds between the sidechain carbons of Leu and mainchain amine groups in Ilamycin C (DBAASPN_14863), Ilamycin E (DBAASPN_14866), and Rufomycins (DBAASPN_22199-22200) produce six-membered delta lactam (δLAC) rings.

In Vancomycin (DBAASPN_21634), ether bonds between the sidechains of beta-oxy tyrosines and phenylglycine form a macrolactam (LAC) ring.

In a few DBAASP peptides (DBAASPN_20988, DBAASPN_20991-20999, DBAASPN_21001, and DBAASPN_21634), sidechains are stapled by a carbon bond, using the two Aryl groups, creating a Biaryl ring (BAR).

Thus, like USCPs, non-ribosomal SCPs are mainly cyclized by amide bonds (about 55% of non-ribosomal SCPs). ESTs are more frequent (about 40% of non-ribosomal SCPs) among other bonds used for cyclization (see Table 3).

#### 3.2.3. Ribosomal Short Cyclic Peptides

Opposed to non-ribosomal SCPs, ribosomal SCPs mainly use Cys residue for cyclization. To create rings, Cys participates in disulfide and sulfide (thioether) bond formation. In over 80% of ribosomal SCPs, rings are formed through the creation of cystines (CST), aminovinyl cysteines (AVC), and lanthionines (LAN). Such rings are not formed in non-ribosomal SCPs (see Table 3).

Peptides with rings, created by amide bonds represent only about 20% of ribosomal SCPs of DBAASP, whereas over half of non-ribosomal SCPs are cyclized through amide bonds. Also, as observed in Table 3, lactone rings (LCNs) are not represented in ribosomal SCPs, while they constitute about 40% of non-ribosomal SCPS.

One additional difference, concerning five-membered rings, is of note. In ribosomal SCPs, five-membered rings (such as OXZ and OXZN) are created by ether bonds, while, in non-ribosomal SCPs, five-membered rings are mainly closed by thioether bonds, forming rings such as THZ, THZN, and TZD. Only THZ is a shared five-membered ring, appearing in both non-ribosomal and ribosomal SCPs.

##### Intrachain Bonds and Created Rings in Ribosomal Peptides

**Figure 4:**
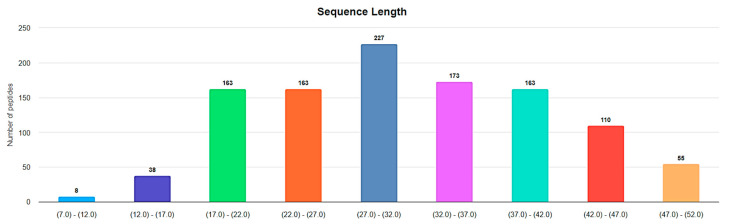
Length distribution of ribosomal disulfide-bonded peptides up to 50 AA in DBAASP. (https://dbaasp.org/statistics?page=physicochemical-statistics, analysis performed on 13 May 2024).

**Disulfide bond**. In the majority of ribosomal SCPs, rings are formed through the disulfide bonds. Figure 4 represents the distribution of lengths for ribosomal disulfide-bonded peptides in DBAASP, ranging up to 50 AA. The total number of such peptides is 1100, which constitutes the majority of all cyclic ribosomal peptides (1269) of that length.

The lengths of approximately 360 ribosomal disulfide-bonded peptides fall within the interval of 6–25 AA in length. That is, they are short. In this section, we will focus on disulfide-bonded short ribosomal cyclic peptides (RSCPs).

**Figure 5:**
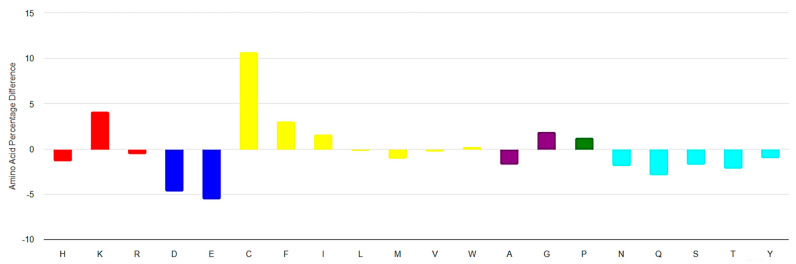
Amino acid composition of ribosomal short cyclic peptides (RSCPs) of length 6–25 AA from DBAASP relative to UniProt. On the *x*-axis, residues are represented in one letter code. Colors: red—positively charged; blue—negatively charged; yellow—hydrophobic; purple—small; green—special; cyan—polar uncharged residues. (https://dbaasp.org/statistics?page=compositional-statistics, accessed on 13 May 2024).

The amino acid composition of the RSCPs from DBAASP, compared to the compositions of UniProt [23] sequences (Figure 5), reveals that RSCPs are more hydrophobic (with a higher abundance of phenylalanine and isoleucine) and more basic (due to the higher abundance of lysine) compared to the ‘average protein’. RSCPs also contain more cysteines and glycines than the ‘average protein’. The presence of glycine is likely related to the requirements for flexibility to close the ring and, at the same time, glycine can promote ‘chameleonic’ ability of a peptide, while the presence of cysteine is crucial for cyclization, which is important for the activity and chemical stability.

**Figure 6:**
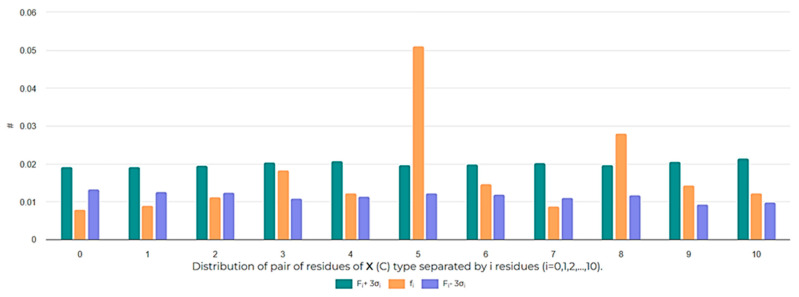
Frequencies of the appearance of *i*-spaced pairs of Cys residues (*i* = 0, 1, 2,…, 10) in disulfide-bonded ribosomal SCPs. The # symbol indicates frequency values.

The distribution of cysteines along the peptide chain is also an interesting aspect to investigate in order to assess the size of cycles that can be closed. The analysis of the distribution of i-spaced amino acid pairs (DiSAAP), specifically focusing on cysteine pairs, in RSCPs of DBAASP reveals a statistically significant abundance of pairs with 5 and 8 AA spaces, while pairs with 0 and 1 spaces are scarce compared to random distribution (Figure 6). These findings can be explained by the role of Cys residues. Cys can be the source of intrachain bonds and, through post-translational modification, it can stabilize certain peptide structures. Taking into account the geometry of the sidechain, it is problematic to form a disulfide bond by pairs of Cys residues spaced by 0 or 1 residues because of steric hindrances [24]. Therefore, the appearance of neighboring Cys pairs or those separated by one residue is limited. On the other hand, the abundance of Cys pairs with 5 and 8 AA spaces suggests the presence of cyclized loops consisting of 7 and 10 AA, characterizing many disulfide-bonded RSCPs in DBAASP. For instance, the abundance of Cys pairs spaced by 5 AA points to the prevalence of ‘Rana box’-containing peptides (RBPs) among RSCPs of DBAASP. RBPs, such as Brevinins (DBAASPR_1009), Nigrocins (DBAASPR_1542), etc., contain small C-terminal loops closed into rings by disulfide bridges and N-terminal linear tails containing more than 7 AA [25]. Consequently, the overall topology of chains of such peptides resembles a lasso. In the DBAASP, peptides with bigger disulfide-bonded loops that differ in amino acid composition from RBPs [26] or that are situated at the N-terminus of peptides [27] can be found.

Moreover, when the linear tail is absent, the peptide chain topology resembles a hairpin. A small size, rigid structure, stability to proteases, and wide spectrum of biological functions make hairpin AMPs an attractive molecular basis for drug design [28]. Many disulfide-bonded RSCPs from DBAASP, such as Protegrins (DBAASPR_672), Tachyplesins (DBAASPR_2229), Arenicins (DBAASPR_2025), Θ-defensins (DBAASPR_823), etc. have hairpin topology of chains.

**Figure 7:**
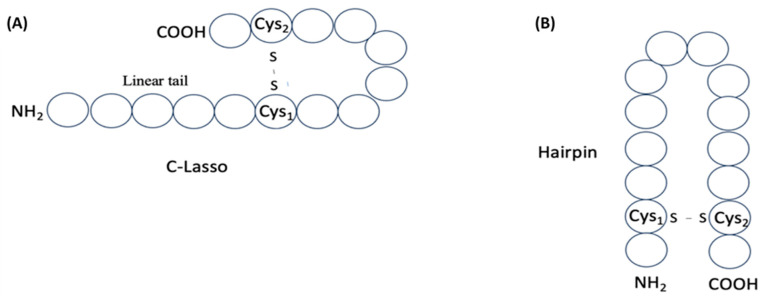
Schematic representation of structures of single disulfide-bond-containing peptides: (**A**) ‘lasso-like’ and (**B**) ‘hairpin-like’. Circles correspond to amino acids.

Thus, among disulfide-bonded ribosomal peptides of DBAASP, about 290 contain a single disulfide bond, allowing us to envision their structure. These peptides can adopt either a ‘lasso-like’ (LL) structure (Figure 7A), with a C- or N-terminal cyclized loop, or a ‘hairpin-like’ (HL) structure, formed when the cysteines are located near the peptide termini (Figure 7B).

To define the type of structure, the parameters *s* and *p* have been introduced. For the given length of peptide *L*, for positions *n*_1_ and *n*_2_ (*n*_1_ < *n*_2_) of first (C_1_) and second (C_2_) cysteines, respectively, the value of *s* and *p* are calculated as:

$$s=\left(L-n_{2}+n_{1}+1\right)/L$$

$$p=\left(L-n_{2}\right)/{(n}_{1}-1)$$

Relying on the values of *s* and *p,* the correspondence of the peptides to LL or HL structure has been assessed. If *s* ≥ 0.5 and 0.3 ≥ *p* ≥ 3 or *n*_1_ = 1, then the peptide structure is defined as LL and, otherwise (when *s* < 0.5), the peptide is defined as HL. In the case of LL peptides, we distinguish N-LL (when *L − n*_2_ > *n*_1_) and C-LL (when *L* − *n*_2_ < *n*_1_). Among ribosomal SCPs that are cyclized by one disulfide bond, the majority have been defined as C-LL and only one as N-LL. The number of HL peptides is about 60 (see Table 4). The size of loops of LL peptides are about 7–8 AA (including Cys-s), while HL loops are a little larger, about 10–11 AA. Such macrocyclic peptides have some conformational freedom and, consequently, flexibility.

**Figure 8:**
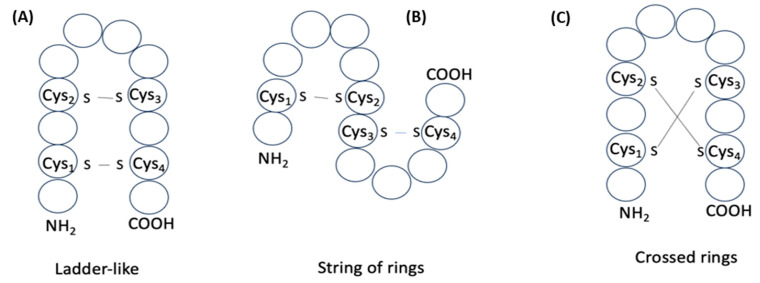
Schematic representation of structures of two disulfide-bond-containing peptides: (**A**) ‘ladder-like’; (**B**) ‘string of rings’; (**C**) ‘crossed rings’. Circles correspond to amino acids.

The structures of about 70 peptides among disulfide-bonded ribosomal SCPs are stabilized by two or more disulfide bonds. Various combinations of pairing can be considered for a given number *n* = 2*k* (*k* = 1, 2, …, *m*) of cysteines (C) that participate in the formation of *k* disulfide bonds. The number (*N*) of variants of pairing to form *k* disulfide bonds is assessed by the formula:

$$N=(2k)!/{(2}^{k}\;k!),$$

while the number of structures created as a result can be classified into three groups (Figure 8). If cysteines of the peptide with *k* disulfide bonds are enumerated according to the increasing positions along the chain as C_1_, C_2_, C_3_, …, C_n_, then pairing schemes that define three different groups of structures can be presented. For example, the pairing of cysteines according to the scheme C_1_ − C_n_, C_2_ − C_n−1_, …, C_n/2_ − C_n/2+1_ (Figure 8A) gives the formation of ‘ladder-like’ multicyclic structures (LD). Another scheme C_1_ − C_2_, C_3_ − C_4_, …, C_n−1_ − C_n_ (Figure 8B) is the formula to form structures consisting of ‘strings of rings’ (SR) closed by disulfide bonds. All other variants of pairing cause the crossing of rings formed by disulfide bonds (Figure 8C) and, thus, the formation of structures with ‘crossed-rings’ (CR).

Consequently, disulfide bonds can create three kinds of multicyclic structures: LD, SR, and CR. For *k* = 2, each kind of structure is presented with a single variant of pairing. With the increasing of *k*, the number of variants of pairing causing CR is increased, while variants that correspond to LD and SR are always equal to one. Therefore, if we consider the process of pairing of cysteines as random, we can suppose that, in disulfide-bonded peptides, CR structures prevail. Indeed, in the majority of peptides in DBAASP with three and more disulfide bonds, pairing of cysteines results in crossing of cycles, which means CR structures are formed, while other types of structures appear rarely (see Table 4). Although, opposed to this, in the short peptides with two disulfide bonds, the LD structure, having a hairpin topology, markedly prevails over the other two types of structure and, thus, this fact cannot be considered as a result of a random process. We can also note non-randomness concerning the fact that C-LL type of structure prevails over N-LL for peptides with one disulfide bond. Apparently, simple structures, like LL and HP, are self-sufficient to perform defense functions. The abundance of Gly and Pro relative to the ‘average protein’ is apparently linked with the necessity to promote such simple structures, which can be the building blocks to form longer and more structurally complex AMPs.

**Thioether bond (sulfide).** DBAASP stores more than 85 ribosomal peptides with thioether bonds, ranging in length from 6 to 38 AA. Peptides are rich in Cys, Ser, and Thr. Thioether bonds are formed between the sidechains of Cys and the sidechains of Thr or Ser (their derivatives), as in lantibiotics [29], or between the sidechains of Cys with the carbonyl group (mainchain) of the neighboring amino acid, as in peptides containing thiazole ring. Thioether bonds predominantly form the amino acid lanthionine (see Table 1) and, consequently, lantibiotics are more widespread among thioether-bond-containing peptides. In lantibiotics, residues participating in sidechain-to-sidechain bonds are spaced by 2–5 AA along the chain and, thus, they form small rings of 4–7 AA (including Cys and Ser or Thr). Such rings are rarely crossed to form CR structures. For example, Nisin contains five rings and, among them, three do not cross each other. Only the fourth and the fifth are crossed. The majority of lantibiotics have one or more inter-crossed rings in their structures. However, there are peptides that contain a string of uncrossed rings, such as Enterocin W-beta (DBAASPR_1475).

Along with the thioether bonds, lantibiotics use disulfide (e.g., Enterocin W-alpha—DBAASPR_1474) and head-to-tail amide bonds (e.g., Subtilosin-A—DBAASPR_6076) for cyclization. Unlike other lantibiotics, Subtilosin-A has a ladder-like structure. The number of rings varies from three to seven.

Another post-translational modification links the sidechains of Cys with the sidechains of Thr or Ser by TIE bonds and results in the creation of aminovinyl cysteine (AVC) rings (Table 1). Often, such rings appear in combination with lanthionine rings. However, there are some peptides containing AVC rings without LAN rings, such as Microvionin (DBAASPR_21973), Goadvionin B2 (DBAASPR_21974), Thioholgamides (DBAASPR_21975 and DBAASPR_21976), JBIR-140 (DBAASPR_21979), and Thioviridamide (DBAASPR_21978). Structures adopted by these peptides resemble LL-type, because the Cys participating in the formation of AviCys is the C-terminal amino acid.

As mentioned above, there is an opportunity to link the sidechain of Cys with the carbonyl group of the neighboring amino acid through a TIE bond to create aromatic, unsaturated thiazole ring (THZ). The number of THZ-containing peptides in DBAASP is not high. Only eight peptides, such as Micrococcins, Patellamides, Bottromycines, etc., have been identified (see Table 3). The structure of peptides containing more than one thiazole ring can be presented as a ‘string of rings’.

Among the peptides with TIE bonds in DBAASP, about 70 have a length of less than 25 AA and can be included in the set of ribosomal SCPs (Table 3).

**Amide bond.** Amide bonds are used more rarely than disulfide bonds to close rings in ribosomal SCPs. A total of 66 SCPs are cyclized into NCB rings, while the number of peptides with LAC rings formed by bonding of sidechain-to-mainchain equals 30 (Table 3). It is worth noting that, although LAC rings in both ribosomal and non-ribosomal SCPs are closed by isopeptide bonds, they are distinguished by the groups of side- and mainchain atoms participating in bond formation. As mentioned above, LAC rings of the non-ribosomal SCPs and USCPs are closed by bonding between the amine group of sidechains of basic residues (such as diaminopropionic acid—DAP, diaminobutyric acid—DAB, Lys, ornithine, 3-aminotetradecanoic acid, 3-aminohexadecanoic acid, etc.) and C-terminal carboxyl groups of the mainchain. In contrast, in the ribosomal SCPs, the amine group of the N-terminus forms an isopeptide bond with the carboxyl sidechain of a glutamic or aspartic acid residues to create an LAC ring of 7–9 AA. Such peptides form a family of ribosomally synthesized and post-translationally modified peptides called ‘lasso’ peptides (LP) [30]. Biosynthetic gene clusters (BGCs) for lasso peptides are presented in many bacterial genomes [31]. Often, the additional ring is formed in the C-terminal tail by disulfide bonds. The C-terminal tail is trapped within the ring either by bulky amino acids or disulfide bridges or both [30]. Of note, due to the frequent appearance of Asp and Glu in LP, they are mainly anionic.

**Amine, imine, ether, ester, and carbonyl bonds.** In addition to disulfide, thioether, and amide bonds, other bonds, such as, amine, imine, ester, and carbonyl, are used to close rings in ribosomal SCPs. However, cyclic peptides with such rings are scarce in DBAASP. As mentioned above, one of the reasons of scarcity could be insufficient data in DBAASP at the moment.

As in the case of non-ribosomal SCPs, in ribosomal SCPs, **amine bonds** are used to create LAC rings. Five such SCPs can be found in the DBAASP. They are Dynobactin A (DBAASPR_20296) and type B lantibiotics of Duramycin (DBAASPR_19104) and Cinnamycin (DBAASPR_21774) families. In contrast to non-ribosomal SCPs, where sidechain-to-mainchain bonding takes place, in ribosomal SCPs, amine bonds link the sidechains of pairs of residues Lys and Ser (by forming Lysinoalanine in lantibiotics, e.g., in Duramicyn B—DBAASPR_19105) or His and Tyr (between the imidazole Nε2 of His and β-carbon of Tyr in Dynobactin A).

LAC rings are created in two SCPs of DBAASP (Bottromycin family, DBAASPR_21130) by bonding of N-terminal amine group nitrogen to the mainchain carbonyl group carbon of 3-methyl-valine through **imine bonds**.

**Carbon bonds** between the sixth carbon of the indole ring of Trp with the β-carbon of another residue (Lys, Asn, or Trp) create an LAC ring in ribosomal SCPs, such as Dynobactin A (DBAASPR_20296) and peptides of the Darobactin family (DBAASPR_17389 and DBAASPR_22221-22222).

In the ribosomal SCP Micrococcin P1 (DBAASPR_21120) [32], an unsaturated six-membered pyrimidine ring is formed by two carbon bonds: between the β-carbons of the first and tenth dehydroalanines (DHA) and between the carbonyl group carbon of the ninth Cys and the α-carbon of the first DHA.

**Ether bonds**, which are not represented in non-ribosomal SCPs, participate in the creation of five-membered unsaturated rings in ribosomal SCPs. SCPs of Patellamide family (e.g., DBAASPR_21121) consists of less aromatic oxazoline and meth-oxazoline rings, while Microcyclamide (DBAASPR_21139) and Wewakazoles (DBAASPR_21267) involve more aromatic oxazole and meth-oxazole. Additionally, ether bonds between the C7 indole carbon of one tryptophan and the β-carbon of another tryptophan form a macrolactam (LAC) ring in post-ribosomally synthesized peptides of the Darobactin family (DBAASPR_17389, DBAASPR_22221-22222; refer to Figure 1 for the structure of Darobactin A).

## 4. An Impact of Cyclization on Peptide Potency

It is generally accepted that cyclization can increase the potency of antimicrobial peptides. It enhances the stability and structural rigidity of AMPs, resulting in improved resistance against enzymatic degradation and better interaction with microbial membranes, often leading to enhanced antimicrobial efficiency in vivo. Moreover, in certain in vitro studies involving Rana-box-containing AMPs [33,34] or disulfide-bonded hairpin peptides [35], doubt is expressed on the key role of cyclization on maintaining the antimicrobial potency. It is important to note that removing the Rana box from AMPs or making substitutions in the peptide sequence alters physicochemical properties of peptides, making it incorrect to compare their potencies under such conditions. To assess the impact of cyclization on peptide potency, it is advisable to compare cyclic and linear peptides with the same amino acid sequence. Furthermore, it is preferable that in vitro assessments of peptide potency intended for comparison are conducted under identical conditions (target bacterial strains, type of culture media, colony forming units (CFU), measures of activity, etc.). Therefore, we believe that the most reliable data for comparison are those obtained in the same laboratory.

In the DBAASP database, data on a series of peptides designed by various laboratories to evaluate the impact of cyclization on peptide activities have been compiled [36,37,38,39,40]. These series consist of pairs of peptides with identical AA sequences but differing in the type of intrachain bonds used in cyclic counterparts. The cyclic counterparts are generated using either disulfide bonds [36,37,38,39] or head-to-tail amide bonds [40].

It is worth noting that, for a reliable interpretation of the comparison results, the peculiarities of the broth microdilution method (MIC assay) used to assess peptide potencies in vitro must be considered. The precision of the MIC assay allows for the differentiation of the potencies of two compared compounds with high reliability only if their MICs differ by more than one dilution (two or more).

**Figure 9:**
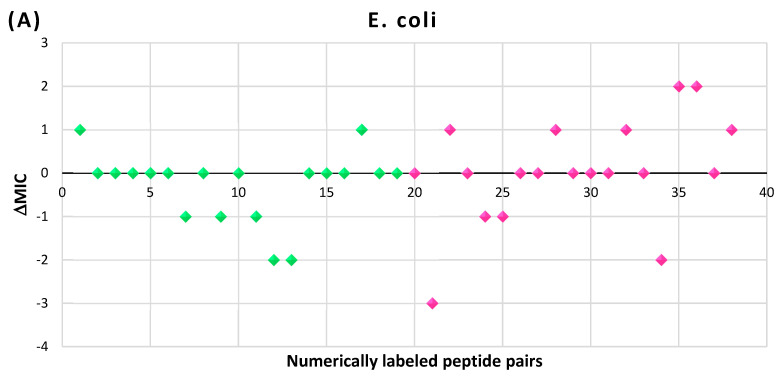

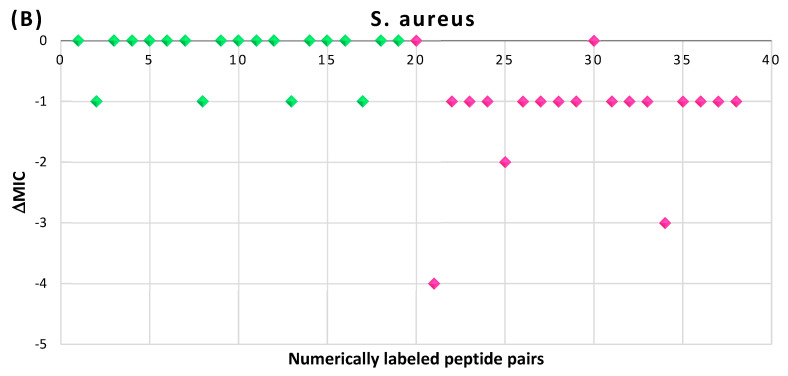
Differences in the MICs (∆MIC) for pairs of cyclic and linear AMPs with identical sequences, expressed in units of dilution: (**A**) tested on *E. coli* strains; (**B**) tested on *S. aureus* strains. Green rhombi—DSB set; pink rhombi—HT set.

In Figure 9, the results of the MIC comparison for the pairs of peptides are presented (raw data can be accessed in Supplementary Materials, Table S1). There are a total of 38 pairs, numerically labeled and arranged along the *x*-axis of Figure 9. The first 19 pairs belong to the disulfide bond set (DSB set), while the remaining 19 pairs belong to the head-to-tail amide bond set (HT set). For the given MICs assessed for the linear (MIC_l_) and cyclic (MIC_c_) peptides, differences in the MICs (∆MIC) have been calculated as:

$$∆MIC=sgn\left(r\right)\left\lfloor \left|r\right|\right\rfloor ,\;where\;r=\log_{2}\left({MIC}_{c}/{MIC}_{l}\right)$$

It is worth emphasizing that the DSB set exhibits more heterogeneity in lengths (ranging from 13 to 24 AA) and chain topology (including both ‘lasso-like’ and ‘hairpin-like’ (including ‘ladder-like’) structures) compared to the HT set. The HT set comprises shorter peptides (6–9 AA) with similar chain topologies (ringed mainchains).

As illustrated in Figure 9A, in the majority of pairs tested on Gram-negative *E. coli* strains, the differences between the MIC values do not exceed one dilution interval (with only a few exceptions), suggesting that the cyclization does not significantly affect the activities of peptides against *E. coli* strains. However, in pairs tested on Gram-positive *S. aureus* strains (Figure 9B), the situation is more complex, making it difficult to draw unambiguous conclusions. The comparison results indicate a decrease in MIC caused by cyclization in both the DSB and HT sets, with the tendency being particularly pronounced for peptides from the HT set. Furthermore, in the majority of cases, the differences do not exceed one dilution interval, falling within the margin of experimental error.

Consequently, we can conclude that the activity assessed on Gram-negative *E. coli* strains is independent of peptide cyclization. However, regarding Gram-positive *S. aureus* strains, cyclization may have some impact in specific cases, particularly for HT-cyclized peptides with lengths ranging from 6 to 9 AA. Nevertheless, overall, this influence is not prominently expressed.

In the case of peptides from the DSB set, the results can be explained by considering the significant role of chain topology in the potency of peptides with certain sequences [35,41]. For instance, it has been shown that the hairpin-like topology of a chain can be stabilized by factors other than disulfide bonding, such as cation-π interactions between aromatic and basic residues [42]. Given that AMPs are rich in aromatic and basic residues, which can support the hairpin topology and thus the potency of the peptide, disulfide bonding may have a negligible effect on potency. Moreover, the absence of intrachain bonds leads to increased flexibility of the AMP, which is often crucial for the antimicrobial efficiency. However, the necessity of disulfide bonding to resist proteases remains clear.

Regarding the shorter peptides from the HT set, it could be speculated that the rigid cyclic structure of peptides holds particular value against the envelopes of Gram-positive strains, where a different mechanism of action operates compared to Gram-negative envelopes. However, further data are required to thoroughly examine this hypothesis.

## 5. Conclusions

DBAASP is a repository of more than 22,000 AMPs, including more than 3900 natural peptides, among which over 2000 peptides are cyclic. Nature offers two ways of synthesis of cyclic peptides: post-ribosomal and non-ribosomal [43,44]. There are 769 non-ribosomally synthesized peptides in DBAASP, including 598 cyclic and small peptides (length ≤ 25 AA). The post-ribosomal method of creating cyclic peptides involves post-translational modifications. The number of post-ribosomally synthesized peptides in DBAASP equals 3192, including 484 small cyclic peptides.

It should be noted that DBAASP exclusively stores data on peptides for which in vitro testing has been performed. Therefore, several kinds of cyclic peptides that have not been tested in vitro cannot be included in the database at the current time. This implies that DBAASP does not cover the entire spectrum of cyclic natural AMPs, and it is presumed that many types of cyclic structures not represented in DBAASP are available and/or will be revealed later. Nevertheless, the volume of DBAASP allows us to understand the level of variability of cyclic structures and the types of intrachain bonds responsible for chain cyclization. Consequently, our aim was to uncover common, statistically reliable tendencies, while some particular facts revealed by analysis should be specified with the influx of new data on cyclic peptides.

The data analysis reveals that both systems of synthesis contribute to the remarkable structural variability of small natural AMPs. Simultaneously, the number of types of bonds providing the variability of cyclic structure is limited (see Table 1). The bonds used to cyclize natural peptides include amide, ester, ether, thioether, amine, imine, and carbon. The majority of rings created during non-ribosomal synthesis are closed by amide (isopeptide) bonds, and amide bonds are also employed for cyclization in post-ribosomal synthesis. Disulfide bonds are predominantly formed during post-ribosomal synthesis (Table 5). Only three fungal peptides with CST rings, two from the Malformin family (DBAASPN_21150 and DBAASPN_21150) and PM181110 (DBAASPN_21712), are synthesized non-ribosomally. Rings cyclized by ester bonds (LCN) are a hallmark of non-ribosomally synthesized peptides (Table 5), which suggests that an effective machinery for post-translational creation of intrachain ester bonds between proteinogenic residues did not evolve. It is worth noting that there are sufficient data to draw reliable conclusions for cyclic peptides where rings are closed by the last three kinds of bonds. However, data on other bonds used by natural cyclic peptides for ring closing are insufficient to reveal common tendencies.

The closing of rings through amide bonds is a shared feature in both non-ribosomal and post-ribosomal synthesis (Table 5). However, the chains and residues participating in bond formation vary depending on whether it occurs in eukaryotes or prokaryotes or how they have been synthesized, non- or post-ribosomally. For instance, peptides with rings closed by head-to-tail amide bonds (NCB rings), which are formed between mainchain atom groups, are synthesized by both eukaryotes and prokaryotes. Meanwhile, sidechain-to-mainchain isopeptide bonds (SMIB) for LAC ring closing are preferentially used by bacteria. Currently, only one exception has been identified: certain species of the fungus genus Aspergillus produce SMIB within the USCPs, such as peptides from the Sclerotiotide family (e.g., DBAASPN_18451). It has to be noted that, in post-ribosomal synthesis, SMIBs mainly involve the sidechain carboxyl of acidic amino acids and N-terminal amine groups. In non-ribosomal synthesis, SMIBs are formed between the sidechain amine group of basic amino acids and the C-terminal carboxyl group.

In general, rings occurring in post-ribosomally and non-ribosomally synthesized cyclic AMPs differ in type, size, participating chains, and bonds used for closing. Consequently, the protein complexes utilized in the two different methods of synthesizing natural cyclic peptides are distinct. However, there are rings that appear in both systems of synthesis used by nature. For instance, NCB rings closed by amide bonds between a mainchain group of atoms or five-membered rings such as thiazole closed by thioether bonds between main- and sidechains appear in both systems of synthesis. Consequently, it is reasonable to suppose that proteins responsible for creating similar rings in the two different systems of synthesis are homologous. However, this supposition has been disproven based on the comparison of the proteins involved in creating the rings in the two systems of synthesis. The comparison reveals that the proteins engaged in the creation of similar rings during post-ribosomal and non-ribosomal synthesis are not homologous. Therefore, the supposition on convergent evolution arises [44].

The requirement for cyclic structure formation has an impact on the amino acid composition of cyclic AMPs. Post-ribosomally synthesized AMPs are enriched with Cys, which forms the basis for the creation of ring-forming amino acids such as cystines and lanthionines. Ultra-short cyclic AMPs are rich in Pro and Gly, amino acids that promote chain turning and ring closing. Some cyclic peptides have a non-traditional (for AMPs) composition of amino acids and utilize acidic residues to close the ring by forming amide bonds with N-terminal amine groups, as seen in lasso peptides.

Rings in small cyclic peptides (≤25 AA) closed by different bonds vary in size and the topology of chains. Rings closed by amide, disulfide, and ester bonds constitute either micro- (e.g., DKP, DSL) or macrolactams. Amine, imine, carbon, thioether, and ether bonds are apparently responsible for creating micro rings such as four-membered (βLAC), five-membered (THZ, TZN, TZND, OXZ, OXZN, and PRL), and six-membered (PYR) rings. The topology of macrolactams, i.e., polypeptide chains closed by bonds such as amide (creating NCB and LAC rings), disulfide (creating CST rings), and ester (creating LCN rings), can adopt four forms: ‘hairpin-like’, ‘lasso-like’, ‘string of rings’, and ‘crossed rings’ structures. The majority of short cyclic ribosomal peptides closing rings by DSB, AMD, and EST bonds adopt hairpin-like or lasso-like structures. These compact yet flexible structures provide membrane permeability, while intrachain bonds ensure chemical stability. Depending on membrane composition, such membrane-active peptides can perturb lipid bilayer, leading to either disruption of membrane or their translocation through it to reach the cytoplasmic target.

We hope that the knowledge of the peculiarities of ring closing in natural peptides revealed in this work will contribute to the design of new cyclic compounds with desired pharmacological features.

## Figures and Tables

**Table 1:** Rings and ring-closing intrachain bonds (RCBs) that formed post-ribosomally or non-ribosomally in small AMPs.

RCBs	Type of Rings (Participating Chains) [Met in Non-Ribosomal (NR) and Ribosomal (R) Cyclic AMPs]			
TIE * 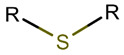	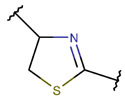 THZN *** (SMB **) [NR]	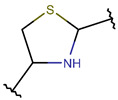 THZD *** (SMB) [NR]	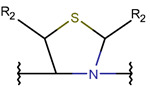 THZD (SSB **) [NR]	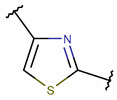 THZ *** (SMB) [NR, R]
	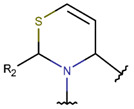 TZ *** (SSB) [NR]	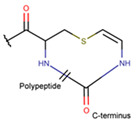 AVC *** (SSB) [R]	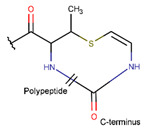 MeAVC *** (SSB) [R]	
	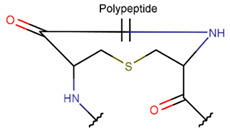 LAN *** (SSB) [R]		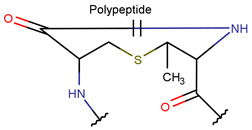 MeLAN *** (SSB) [R]	
ETH * 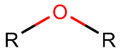	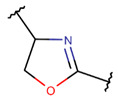 OXZN *** (SMB) [R]	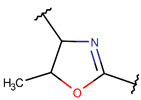 MeOXZN *** (SMB) [R]	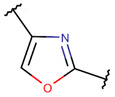 OXZ *** (SMB) [R]	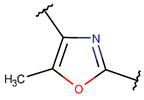 MeOXZ *** (SMB) [R]
	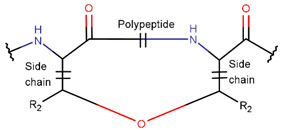 LAC *** (SSB) [NR, R]			
EST * 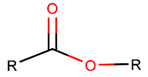	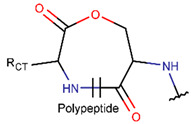 LCN *** (SMB) [NR]		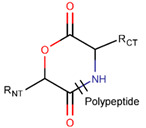 LCN (MMB **) [NR]	
DSB * 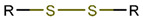	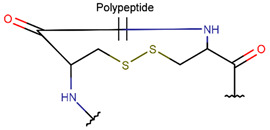 CST *** (SSB) [NR, R]		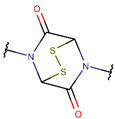 DSL *** (SSB) [NR]	
AMD * 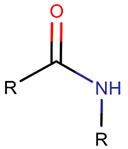	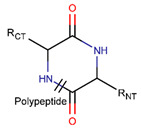 NCB *** (MMB) [NR, R]		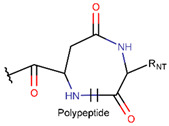 LAC (SMB) [R]	
	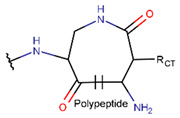 LAC (SMB) [NR]		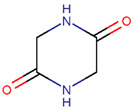 DKP *** (MMB) [NR]	
IMN * 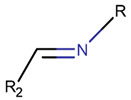	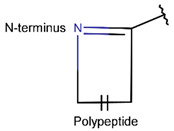 LAC (MMB) [NR]			
AMN * 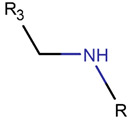	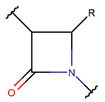 βLAC *** (SMB) [NR]	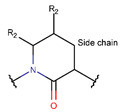 δLAC *** (SMB) [NR]	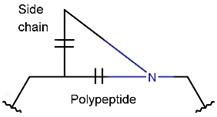 LAC (SMB) [NR]	
	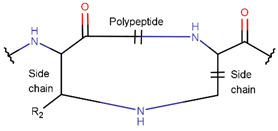 LAC (SSB) [R]		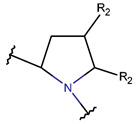 PRL *** (SMB) [NR]	
CAR * 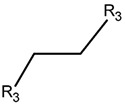	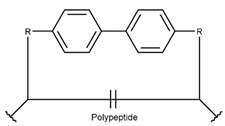 BAR *** (SSB) [NR]		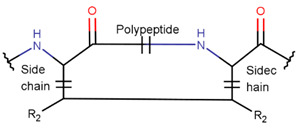 LAC (SSB) [R]	
	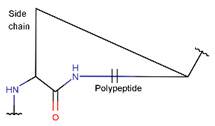 LAC (SMB) [R]		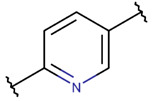 PYR *** (MMB, SSB) [R]	

* RCBs: AMD—amide; AMN—amine; IMN—imine; CAR—carbon; ETH—ether; TIE—thioether (sulfide); DSB—disulfide; EST—ester. ** Participating chains: SMB—sidechain–mainchain bond; MMB—mainchain–mainchain bond; SSB—sidechain–sidechain bond. *** Types of rings: THZN—thiazoline (cyclic imino thioethers having an unsaturated five-membered structure, 1 double bond); THZD—thiazolidine (cyclic amino thioethers having a saturated five-membered structure); THZ—thiazole (cyclic imino thioethers having an unsaturated five-membered structure, 2 double bonds); TZ—thiazine (cyclic imino thioethers having an unsaturated six-membered structure, 2 double bonds); OXZN—oxazoline (cyclic imino ethers having an unsaturated five-membered structure, 1 double bond); OXZ—oxazole (cyclic imino ethers having an unsaturated five-membered structure, 2 double bonds); MeOXZN—methyloxazoline; MeOXZ—methyloxazole; LCN—macrocyclic ester, lactone; LAC—macrocyclic amide, macrolactam; βLAC—cyclic amide, β-lactam; δLAC—cyclic amide, δ-lactam; DKP—cyclic amide, head-to-tail cyclized dipeptide, 2,5-diketopiperazine; NCB—macrocyclic amide, head-to-tail cyclized macrolactam; CST—macrolactam closed by cystine; DSL—disulfide linkage between two C^α^; LAN—macrolactam closed by lanthionine; MeLAN—macrolactam closed by methyllanthionine; BAR—macrolactam stapled by biaryl group; PYR—pyridine (a benzene core in which one -CH group is replaced by a nitrogen atom); PRL—cyclic secondary amine, pyrrolidine; AVC—aminovinyl cysteine; MeAVC—aminovinyl methyl cysteine. R_x_—‘R’ denotes an atom or group of atoms and ‘X’ denotes their number; R_CT_—sidechain of C-terminal amino acids; R_NT_—sidechain of N-terminal amino acids. Analysis performed on 13 May 2024.

**Table 2:** The numbers of ultra-short cyclic peptides (USCPs) with defined rings, ring-closing bonds (RCBs), and lengths.

Type of Synthesis	Peptide Length	Number of Peptides											
		Total	Total Cyclic	Certain RCBs, Participating Chains and Types of Rings									
				AMD * MMB ** NCB ***	AMD MMB DKP ***	AMD SMB ** LAC ***	AMN * SMB βLAC ***	AMN SMB PRL ***	DSB SSB CST ***	DSB * SSB ** DSL ***	EST * SMB LCN ***	TIE* SSB THZD ***	TIE SSB TZ ***
Non-Ribosomal	2	93	89	0	79	0	4	2	1	1	6	3	1
	3	47	32	13	3	12	0	0	0	0	4	0	0
	4	55	43	31	0	0	0	0	0	0	10	0	0
	5	68	61	21	0	0	0	0	2	0	39	0	0
Post-Ribosomal	2	0	0	0	0	0	0	0	0	0	0	0	0
	3	0	0	0	0	0	0	0	0	0	0	0	0
	4	1	1	1	0	0	0	0	0	0	0	0	0
	5	6	1	1	0	0	0	0	0	0	0	0	0

* RCBs: AMD—amide; AMN—amine; TIE—thioether (sulfide); DSB—disulfide; EST—ester. ** Parcipating chains: MMB—mainchain–mainchain bond; SMB—sidechain–mainchain bond; SSB—sidechain–sidechain bond. *** Types of rings: NCB—macrocyclic amide, head-to-tail cyclized macrolactam; DKP—cyclic amide, head-to-tail cyclized dipeptide; LAC—macrocyclic amide, macrolactam; βLAC—cyclic amide, β-lactam; PRL—cyclic secondary amine, pyrrolidine; CST—macrolactam closed by cystine; DSL—disulfide linkage between two C^α^; LCN—macrocyclic ester, lactone; THZD—thiazolidine; TZ—thiazine. Analysis performed on 13 May 2024.

**Table 3:** The numbers of natural short cyclic peptides (SCPs) with defined rings and ring-closing intrachain bonds (RCBs) formed post-ribosomally or non-ribosomally.

RCBs	Participating Chains	Types of Rings	Non-Ribosomal	Post-Ribosomal
DSB *	SSB **	CST ***	0	360
TIE *	SSB	LAN ***	0	22
		MeLAN ***	0	22
		AVC ***	0	12
		MeAVC ***	0	5
	SMB **	THZ ***	4	8
		THZN ***	4	0
		THZD ***	1	0
AMD *	MMB **	NCB ***	138	66
	SMB	LAC ***	66	30
AMN *	SMB	LAC	8	0
		δLAC	4	0
	SSB	LAC	0	5
EST *	SMB	LCN ***	142	0
ETH *	SMB	OXZ ***	0	4
		MeOXZ ***	0	5
		OXZN ***	0	2
		MeOXZN ***	0	1
	SSB	LAC	3	1
IMN *	MMB	LAC	0	2
CAR *	SMB	LAC	0	6
	SSB	LAC	0	4
		BAR ***	12	0
		PYR ***	0	1
	MMB	PYR	0	1
Total Number			367	482

* RCBs: DSB—disulfide; TIE—thioether (sulfide); AMD—amide; AMN—amine; EST—ester; ETH—ether; IMN—imine; CAR—carbon. ** Parcipating chains: SSB—sidechain–sidechain bond; SMB—sidechain–mainchain bond; MMB—mainchain–mainchain bond. *** Types of rings: CST—macrolactam closed by cystine; LAN—macrolactam closed by lanthionine; MeLAN—macrolactam closed by methyllanthionine; AVC—aminovinyl cysteine; MeAVC—aminovinyl methyl cysteine; THZ—thiazole; THZN—thiazoline; THZD—thiazolidine; NCB—macrocyclic amide, head-to-tail cyclized macrolactam; LAC—macrocyclic amide, macrolactam; δLAC—cyclic amide, δ-lactam; LCN—macrocyclic ester, lactone; OXZ—oxazole; MeOXZ—methyloxazole; OXZN—oxazoline; MeOXZN—methyloxazoline; BAR—macrolactam stapled by biaryl group; PYR—pyridine. Analysis performed on 13 May 2024.

**Table 4:** The types of structures of disulfide-bonded short cyclic peptides (SCPs).

Number of DSBs	Structural Types				
	C-LL	N-LL	HP	LD	CR
1	227	1	60	NA	NA
2	NA	NA	29	29	8
3	NA	NA	3	3	10
4	NA	NA	4	4	16

C-LL and N-LL—C- and N-terminally looped ‘lasso-like’ structures, respectively; HP—‘hairpin-like’; CR—crossed rings; LD—‘ladder-like’; NA—not appeared; LD can simultaneously be considered as HP.

**Table 5:** The numbers of small peptides synthesized post-ribosomally or non-ribosomally in specific source organisms cyclized by amide, disulfide, and ester intrachain bonds (RCBs).

Type of Synthesis		Non-Ribosomal				Post-Ribosomal			
RCBs		AMD *		DSB *	EST *	AMD		DSB	EST
Participating Chains		MMB **	SMB **	SSB **	SMB	MMB	SMB	SSB	SMB
Types of Rings		NCB ***	LAC ***	CST ***	LCN ***	NCB	LAC	CST	LCN
Sources	Animalia	33	0	0	18	18	0	931	0
	Plantae	3	0	0	1	139	0	238	0
	Protista	0	0	0	0	0	0	2	0
	Fungi	74	10	3	61	0	0	24	0
	Bacteria	88	66	0	122	19	32	38	0
	Archaea	0	0	0	2	0	0	1	0

* RCBs: AMD—amide; DSB—disulfide; EST—ester. ** Participating chains: MMB—mainchain–mainchain bond; SMB—sidechain–mainchain bond; SSB—sidechain–sidechain bond. *** Types of rings: NCB—macrocyclic amide, head-to-tail cyclized macrolactam; LAC—macrocyclic amide, macrolactam; CST—macrolactam closed by cystine; LCN—macrocyclic ester, lactone. Analysis performed on 13 May 2024.

## Data Availability

The data presented in this study are openly available in the DBAASP database at https://www.dbaasp.org and are included in the article/Supplementary Materials.

## Supplementary Materials

The following supporting information can be downloaded at: https://www.mdpi.com/article/10.3390/ph17070845/s1, Table S1 contains the raw data corresponding to Figure 9.
