# Supplementary material for: Small Natural Cyclic Peptides from DBAASP Database
Source: Pharmaceuticals (Basel). 2024 Jun 27;17(7):845. doi: 10.3390/ph17070845 (PMC11279581; doi:10.3390/ph17070845)
Supplement: Supplementary file 1 [file pharmaceuticals-17-00845-s001.zip › pharmaceuticals-3045380-supplementary.pdf]

Table S1

Pair numbers	DBAASP IDs of the pairs of peptides with the same sequences		Δ MIC	
	**Linear**	**Cyclic**	**for E. coli**	**for S. aureus**
1	16214	16213	1	0
2	16216	16215	0	-1
3	16218	16217	0	0
4	16222	16221	0	0
5	16226	16225	0	0
6	16228	16227	0	0
7	16230	16229	-1	0
8	16232	16231	0	-1
9	16234	16233	-1	0
10	9490	850	0	0
11	1063	8584	-1	0
12	1090	1082	-2	0
13	6209	6208	-2	-1
14	13651	6840	0	0
15	13186	13185	0	0
16	16206	16205	0	0
17	16208	16207	1	-1
18	16210	16209	0	0
19	16212	16211	0	0
20	19712	19713	0	0
21	19716	19715	-3	-4
22	19719	19718	1	-1
23	19722	19721	0	-1
24	19723	19724	-1	-1
25	19725	19726	-1	-2
26	19727	19728	0	-1
27	19736	19737	0	-1
28	19738	19739	1	-1
29	19740	19741	0	-1
30	19742	19743	0	0
31	19744	19745	0	-1
32	19746	19747	1	-1
33	19748	19749	0	-1
34	19752	19751	-2	-3
35	19753	19754	2	-1
36	19756	19757	2	-1
37	19758	19759	0	-1
38	19760	19761	1	-1

The first 19 pairs represent disulfide-bonded peptide pairs (DSB set), the other 19 pairs – N-C-bonded peptide pairs (NCB set);
For the given MICs assessed for the linear (MIC_l) and cyclic (MIC_c) peptides, differences in the MICs (ΔMIC) have been calculated as $\Delta MIC = sgn(r)\lfloor |r| \rfloor$, where $r = \log_2(MIC_c / MIC_l)$.
